# Treatment of Femoral Shaft Pseudarthrosis, Case Series and Medico-Legal Implications

**DOI:** 10.3390/jcm11247407

**Published:** 2022-12-14

**Authors:** Giuseppe Basile, Stefania Fozzato, Quirino Alessandro Petrucci, Mario Gallina, Luca Bianco Prevot, Riccardo Accetta, Simona Zaami

**Affiliations:** 1 IRCCS Orthopedic Institute Galeazzi, 20161 Milan, Italy; 2 Department of Anatomical, Histological, Forensic and Orthopedic Sciences, “Sapienza” University of Rome, 00161 Rome, Italy

**Keywords:** pseudarthrosis, non-union, femoral shaft fractures, medical professional liability, bone graft

## Abstract

Pseudarthrosis (PSA) is a possible complication of femoral shaft fracture treatment. It is often associated with reduced bone quality and can, therefore, adversely affect quality of life. Its treatment poses a major challenge for orthopaedic surgeons. Several authors have set forth different surgical approaches for the treatment of pseudarthrosis, such as internal fixation with plate and screws, replacement of an intramedullary nail or prosthetic replacement. In cases associated with bone loss, osteopenia, or comminution of fracture fragments, autologous or homologous bone grafts may also be used. The chronic outcomes of the surgical treatment of femoral shaft pseudarthrosis, even when consolidation is achieved, are linked to disabling sequelae of clinical-functional relevance, deserving an adequate medico-legal evaluation. The purpose of this retrospective study is to analyse a clinical case series of patients treated for atrophic femoral shaft pseudarthrosis at the IRCCS Orthopaedic Institute Galeazzi, Milan, Italy, from 2014 to 2020 and their orthopaedic-traumatological and medico-legal implications.

## 1. Introduction

Pseudarthrosis (PSA) has been defined in different ways in the literature. It has been described as a lack of radiographic consolidation at six months after the traumatic event and is associated with the necessity for revision surgery. According to the Food and Drug Administration (FDA), the diagnosis of PSA is made after at least nine months from the trauma with no visible signs of progressive healing for three months [1,2].

A higher incidence of PSA has been observed in femoral shaft fractures in recent years. However, it is an infrequent complication compared with pseudarthrosis of the humeral shaft [1], whose incidence is approximately 1.9–10% of all fractures occurring annually [1] or compared with PSA in tibial fractures that are about 13.3% [3,4]. The increased incidence of PSA in femoral fractures, whose incidence is approximately 1.9–5% of all fractures occurring annually [1,5], is due to the increased survival of patients with multiple severe injuries and the broadening of indications for intramedullary nailing [6].

Numerous techniques have been proposed for the treatment of femoral shaft pseudarthrosis, including electromagnetic fields [7], low-intensity ultrasound [8], extracorporeal shock wave therapy (ESWT) [9], external fixators [10] and osteosynthesis with plate and screws [11]. Moreover, several studies have reported positive results after the treatment of non-union of the femur with single or double plate fixation combined with autologous bone graft [12,13].

Undoubtedly, the major severity of the conditions of road polytrauma patients and the increase in the average survival age have led to therapeutic problems due to the greater complexity of fractures and associated comorbidities such as obesity, diabetes, Chronic Obstructive Pulmonary Disease (COPD), correlated visceral injuries, and neurological diseases, including the pathological consequences of the recent severe acute respiratory syndrome coronavirus 2 (SARS CoV-2) pandemic [14].

In the 1970–1980s, femoral shaft PSA was considered an exceptional complication, while delayed union was less rare, affecting open fractures, especially with bone loss and infection. In closed fractures, treated conservatively, a muscle flap was sometimes interposed between the fragments, which constituted an insuperable barrier for the repairing tissue. Furthermore, it was believed that the non-consolidation was caused by an insufficient period of immobilisation in the plaster cast and/or by premature weight-bearing loading and walking [15].

In the past, femur PSA was believed to be mostly related to errors in the treatment method. Recent research has shown that non-union, both atrophic and hypertrophic, is related to a certain degree of vascularisation and mesenchymal stem cells (MSCs) with osteogenic capacity.

The non-consolidation of a femur fracture causes pain, especially during loading, altered and excessive motility at the fracture focus, functional limitations of the contiguous joints and muscle hypotrophy. It can therefore cause serious damage to the “functioning” capabilities of the injured lower limb, with consequent qualitative and quantitative alterations of everyday life activities. The lack of bone continuity of the femoral shaft and the consequent preternatural motility with instability to biomechanical stress prevent lower limb loading and severely reduce active and passive mobility, as well as walking. According to the disability model of the International Classification of Functioning, Disability and Health (ICF), adopted by the World Health Organization (WHO), five levels of disability involving the lower limbs have been identified: level 0 interference with the function is not demonstrable; level 1 interference is associated with vigorous exercise; level 2 analgesic claudication is presented and limits the distance travelled or requires the constant and regular use of an orthosis or a cane; level 3 presence of analgesic claudication with constant use of two sticks associated with orthoses; level 4 is impossible to walk [16]. By adopting these criteria, femoral PSA causes level 3 or 4 disability, with severe or total functional impotence of the lower limb. To this damage are added the deformities caused by the frequent displacement of the fracture’s fragments, the dysmetria, the severe hypotonia from non-use, the joint stiffness of the knee and, to a lesser extent, of the hip [17]. In order to minimise complications, surgical and non-surgical treatment of femoral fracture must be adapted differently for each patient, considering the biological and biomechanical basis of bone healing. Over the past decade, the “diamond concept” for fracture healing has ascribed equal importance to mechanical stability and the biological environment, offering a new paradigm for complex fractures and the management of compromised joints [18,19]. This article has been conceived to elaborate on a series of clinical cases of atrophic pseudarthrosis after femoral shaft fracture treated by our team and to highlight the possible medico-legal repercussions. In fact, the complexity of these injuries can severely impact the ergonomic efficiency of the entire limb, resulting in possible postural alterations, with all the relative repercussions on the overall compromised structure of the patient.

## 2. Materials and Methods

This study is centred on 16 patients, both male and female, with atrophic PSA after femoral shaft fracture, treated at the IRCCS Orthopaedic Institute Galeazzi, Milan, Italy, between 2014 to 2020. Patient charts, operation, and follow-up notes were retrospectively analysed.

The indication for revision surgery was given in case of rupture of the fixation tools, severe osteopenia, cortical bone defect of ≥2 cm at the site of PSA or in the presence of disabling clinical symptoms.

Surgical treatment of atrophic PSA of the femoral stem requires complete removal of the interposed necrotic tissue, remodelling of bone fragments and the restoration of fracture stability. Plate osteosynthesis, applied with an axial compression mechanism, is the best choice for most surgeons. The application of cortico-spongy grafts can increase the likelihood of consolidation. The graft, harvested from the patients themselves (autologous) or from a donor (homologous), enhances the mechanical stability of the synthesis to ensure total immobility of the fragments; in some cases, two plates mounted in parallel can be used for the treatment of humeral shaft PSA, although it may determine increased soft tissue dissection and a higher risk of bleeding. Open reduction and internal fixation with a long compression plate combined with homologous bone grafting is our treatment of choice, even in osteoporosis patients with reduced bone quality. However, in selected cases previously treated with an intramedullary nail, we have considered dynamisation of the nail when possible or replacement of the intramedullary nail after further reaming the femoral canal. Only in case of impossibility to perform a revision of the surgical synthesis has prosthetic replacement been considered.

Healing after the second surgery was radiographically assessed through the presence of a callus in the PSA site with the restoration of bone continuity and through a physical and clinical examination to determine the solidity of consolidation.

As preoperative antibiotic prophylaxis, cefazolin 2 g was administered intravenously, except for two patients who were given clindamycin 600 mg intravenously due to allergy concerns. Antibiotic prophylaxis was continued until the fifth postoperative day.

Given the attention to residual haemostasis at the end of the surgical revision, we never used surgical drains. The surgical wound dressing was renewed every two days.

The rehabilitation of the lower limb muscles started with isometric exercises and passive mobilisation of the affected limb the day after the surgery. Some cases may even call for pharmacological interventions (e.g. the administration of anabolic steroids) [20,21].

An articulated knee brace with long splints (DonJoy X-Act ROM Knee Orthosis^®^, DJO global^®^, Lewisville, TX, USA) was placed after surgery in all the patients for thirty days. The 20% partial weight bearing started at four weeks after surgery. At eight weeks, in the presence of radiographic signs of healing, such as the appearance of normal osteo-reparative phenomena at the level of the pseudarthrosis focus, the patient was granted a tolerance load with two crutches.

Until complete weight bearing was reached, the patient was advised to use vascular elastic stockings associated with the administration of low molecular weight heparin.

All patients underwent periodic clinical and radiographic follow-ups. All data were collected through a retrospective review of patient medical records and radiographs, and a minimum follow-up of 6 months after definitive surgery was requested.

Informed consent was obtained from all patients. The nature of the study did not require approval from the institute’s ethics committee.

## 3. Results

The sample consisted of ten men and six women, with an average age of 50 years (range 12–82) at the time of trauma, which has been initially treated with an intramedullary nail or plate and screws. Nine cases involved the left side and seven the right side. In all cases, atrophic PSA resulted after a fracture caused by a high-energy trauma, mainly a car or motorcycle event.

In our series, eight PSA occurred at the proximal third of the femoral shaft, four at the middle third and four at the distal third. Two patients were obese (body mass index ≥30), four were polytrauma patients with multiple district involvement, one patient had type 1 neurofibromatosis, four patients had multiple comorbidities (diabetes, COPD, and arterial hypertension), three patients were smokers, while none had alcohol abuse issues. In one case, the femoral fracture was linked to a point exposure, classified as type 1 according to the Gustilo-Anderson classification.

Eight patients were treated by intramedullary nail, and eight patients by plate and screws. Two patients had undergone an intermediate surgery to dynamise the medullary nail, previously implanted, in order to address the consolidation delay.

All these procedures were performed in other hospitals and came to our observation after finding the non-union of the fracture.

All patients considered underwent revision surgery with new internal fixation.

The time between the first osteosynthesis and the revision surgery for PSA was, on average, 454 days (range, 302–694 days).

Seven patients experienced the rupture of the implants previously implanted, while in the remaining nine, the implants were still intact when removed by revision surgery. All patients were treated by the same surgeon and the mean intervention time was 163 min (range 40–236).

**Figure 1:**
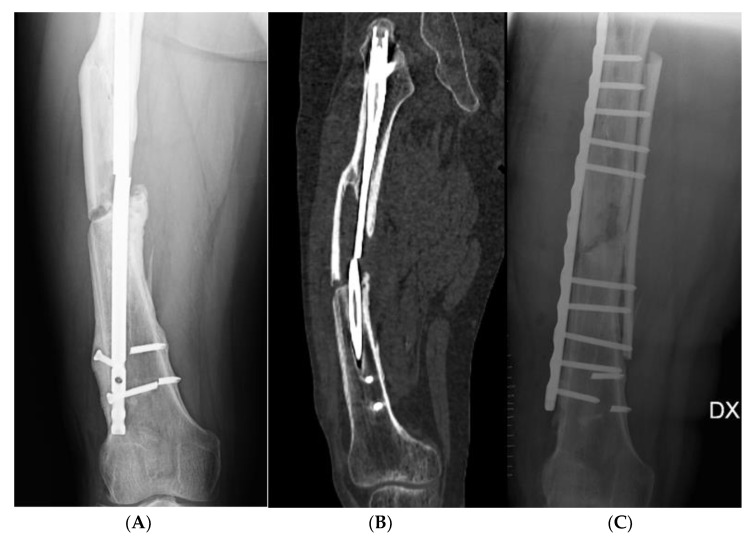
46-year-old patient with a closed 32-B2 fracture. (**A**) Anteroposterior X-ray showing PSA at the middle third of femoral diaphysis, initially treated with an intramedullary nail, which appear broken at the fracture site. (**B**) Coronal CT section of the femur showing the PSA focus, the broken nail and posterior bone loss. (**C**) Anteroposterior X-ray of the femur after revision surgery with lateral plate and screws and medial bone graft splint.

Seven patients were treated with dedicated plate and screws (NCB^®^ plate, Zimmer Inc., Warsaw, IN, USA) associated with homologous bone graft, using a standard lateral surgical approach to the femoral shaft. After the removal of the previous implants, the focus of pseudarthrosis was exposed, removing the fibrous tissue between the bone fragments, and the bone stumps were reduced under fluoroscopic control with a C-arm. A bone graft splint was applied as a medial buttress, and internal fixation was performed with a plate and screw at the lateral side. The anatomical planes were then sutured before applying a sterile dressing (Figure 1).

In three patients, the same surgical approach was implemented, but instead of a bone graft splint, an autologous cortico-cancellous bone graft taken from the iliac crest was placed medially at a non-union level, along with lateral plate and screws fixation.

Two patients were treated by revisioning the intramedullary nail using an antegrade nail with a larger diameter (Gamma^®^ 3 model, Stryker, Kalamazoo, MI, USA) after further reaming of the medullary canal.

**Figure 2:**
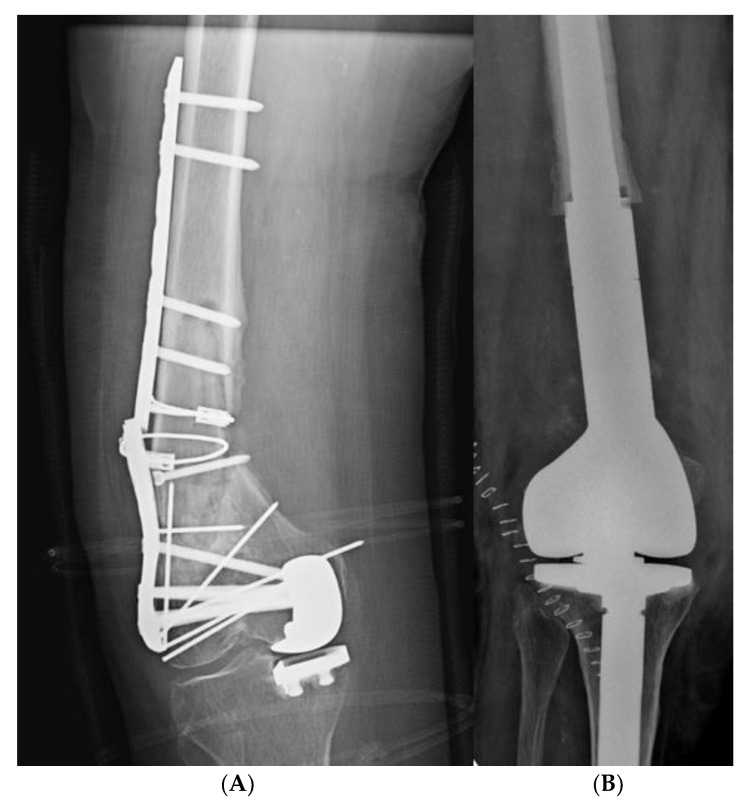
80-year-old patient with 33-A2 femur fracture. (**A**) Anteroposterior X-ray of the femur showing a PSA is present in the distal third of the femoral diaphysis, associated with mono-compartmental knee prothesis, broken plate, multiple Kirshner wires and metal cerclages. (**B**) Anteroposterior knee X-ray showing knee mega prosthesis implantation as revision surgery.

In two patients with PSA of the proximal third of the femoral diaphysis, an anterolateral approach to the hip was performed, with a total hip replacement (Accolade^®^ II model, Stryker, Kalamazoo, MI, USA). In two patients, the onset of non-union in the distal third of the femoral diaphysis was associated with significant bone resorption and marked arthritic degeneration of the knee cartilage. In these cases, it was not possible to proceed with internal fixation, but it was necessary to implant a knee mega prosthesis (LINK^®^ MEGASYSTEM-C^®^ Tumor and Revision System, Waldemar Link GmbH & Co. KG, Hamburg, Germany) (Figure 2).

In all cases, during the revision surgery, a lower level of callus formation at the PSA site was found intraoperatively, compatible with atrophic pseudarthrosis, and probably due to excessive rigidity with a lack of stimulus for the callus formation. The presence of thin shoots of loose fibrous tissue in the interfragmentary space was associated with the loss of bone substance of the fracture stumps, which also appeared sclerotic. The mean follow-up was of 15.4 months (range 6–38). The demographic characteristics of the patients considered and the different treatments applied in our study are summarised in Table 1.

Recovery from pseudarthrosis was reported in 13 patients (81% of cases), including patients who had had total hip arthroplasty and mega prosthesis of the knee implants.

In three patients, the normal reparative healing processes did not occur in the follow-up period. These patients had multiple comorbidities, such as type 2 diabetes mellitus, hypertension, and COPD. No patient had a history of infection at the fracture site, so all the PSA were considered non-septic. There were no postoperative infections after revision surgery in any patient, although some complications did occur. In the postoperative course, four patients were transfused with concentrated red blood cell units for post-surgical anaemia. A supra-fascial fluid collection with no signs of infection was observed in one patient, while another one was left with a dystrophic scar, adhering to the underlying anatomical planes.

Magnetotherapy was used by five patients to stimulate the bone healing of the PSA focus, leading to a progression of osteo-repairing phenomena.

Recovery from pseudarthrosis occurred in all five patients (100%) initially treated with an intramedullary nail and subsequently treated with plate and screws and bone graft implant.

Patients who achieved PSA consolidation after neo-osteosynthesis experienced mild thigh and knee pain, muscle hypotonia of varying degrees, reduced knee range of motion (ROM) with flexion block between 90–110°, and varying degrees of disability. The level of disability was grade 1 in seven cases and grade 2 in two cases.

One patient treated with knee mega prosthesis presented decreased joint ROM, with a deficit of 15° both in flexion and in extension compared with the contralateral knee, and chronic pain, which increased with load and active mobilisation. The other patients treated with knee or hip resection prosthesis had a normal postoperative course, with normal or moderately reduced ROM, occasional pain, mild hypotonia, and 1–2 degree of disability.

During the follow-up, none of the patients showed intolerance to the fixation implants or their mobilisation and in no case was the implanted material removed.

## 4. Discussion

Pseudarthrosis is a possible complication of femoral shaft fractures that require adequate therapeutic planning, which needs to be aimed towards two fundamental goals: mechanical stability to allow the early recovery of joint function and walking, and protection of vascularity, to minimise trophic damages, secondary to excessive cruentation at the PSA focus. Definitive surgery must ensure adequate stability of the fracture site, as well as accurate bone alignment, to allow for optimal functionality of the operated limb.

The ultimate objective is to reduce the risk of permanent disability, often severe in a patient affected by PSA, especially in young patients incurring such a complication after a high-energy trauma.

About possible treatments for PSA, Kouzelis et al. have demonstrated that reaming the femoral canal has an important osteo-inductive biological effect due to reaming debris, which acts as an autologous endogenous graft [22]. Another possible treatment is an internal fixation with plate and screws, a more invasive surgery which allows excision of the fibrous tissue at the PSA focus, direct visualisation of the fracture site, and correction of rotation and mechanical axis [23].

Internal fixation of the plate and screw, however, may not be enough if associated with osteopenia, comminute fracture, or loss of bone stock. High complication rates have been reported in such cases, including misalignment, reduced mobility and persistent non-union [24]. For this reason, the use of bone grafts is increasingly associated with internal fixation with a plate to give more stability to the bone, stimulate the normal healing processes and offset bone loss. Chapman and Finkemeier proved the healing in eighteen patients suffering from supracondylar pseudarthrosis of the femur after fixation with plate and screws and bone graft [25]. Wang and Weng treated 13 patients with removal of previously implanted fixation media, internal fixation, and autograft, reaching a 100% union rate [26]. Maimaitiyiming et al. used dual plate and bone grafting in 14 patients with PSA after femoral shaft fractures, achieving healing in all operated patients [27].

In other cases, as reported by Raju Vaishya et al., the unsuccessful internal fixation of distal femoral shaft fractures, associated with bone loss and arthritis of the knee with or without ligament instability, can be treated with a cemented mega prosthesis, especially in elderly patients [28].

Earlier studies have presented results comparable with our own. To treat femoral shaft PSA, we often use internal fixation with plate and screws associated with the medial bone graft. This surgery allows for the removal of all the fibrotic tissue interposed. It enables the establishment of a stable, but at the same time flexible, system for adequate bone tissue formation. The use of autologous and homologous bone grafts constitutes a potentially invaluable tool; it makes it possible to rely on growth factors stimulating the formation of the reparative callus, thus obtaining a further stabilisation of the PSA focus, considering that patients with such a condition usually have poor bone quality.

However, surgery must always be carefully planned based on fracture type, patient age, the site of the fracture and bone conditions in order to obtain good results both in terms of the healing process and quality of life.

The examination of the proposed cases and the scientific literature allows us to exhibit some orthopaedic-traumatic and medico-legal remarks.

The fracture healing process is influenced by many potentially interfering biological factors. For example, factors related to bone biology and genetic predisposition, patient-related factors such as lifestyle habits and comorbidities and some related to fracture characteristics such as topography or soft tissue injuries [29].

Patient age is very important in determining the recovery process: in children and young adults, the periosteum is rich in osteoblasts and is well vascularised, whereas elderly patients usually exhibit a partially fibrous periosteum, associated with slower callus formation [30].

In patients with osteoporosis, delayed expression of estrogen receptors was detected during the healing process and is associated with reduced callus formation. Nikolaou et al. reported the average consolidation time of femoral shaft fractures treated with a medullary nail in patients with osteoporosis to be three weeks longer than in healthy patients [31]. Furthermore, fracture surgical fixation procedures in patients with osteoporosis are known to incur higher rates of complications, such as loss of reduction, failure implantation, delayed union, or non-consolidation.

The activity of osteoprogenitor cells is also influenced by genetic factors. In fact, the recruitment, differentiation, and proliferation of osteogenic cells during the initial phase of fracture healing are reduced due to the low production of growth factors, such as BMPs and the qualitative and quantitative deficit of MSCs [32,33]. Manigrasso and O’Connor, in an experimental study on rats, have identified several genes, such as C57BL/6, DBA/2 and C3H9, capable of influencing the fracture consolidation process. In mice with C57BL/6 genes, the consolidation of the fracture was achieved in a shorter time [34]. Hofmann et al. have shown reduced cell viability and downregulated gene expression of signalling molecules (IGF-, TGF-β- and FGF) in osteoblasts of patients with hypertrophic bone non-union [35]. It has been reported that polymorphisms and genetic profiles of specific molecules affect both cell differentiation and the generation process of endochondral callus [36]. Such factors may indeed take on increasingly great relevance as personalized medicine based on the unique genomic traits of each individual patient makes further progress [37].

In addition, there is no discounting the role plaid by lifestyle habits such as smoking, alcohol and non-steroidal anti-inflammatory drugs (NSAIDs) abuse, stress, and poor eating habits, which constitute noteworthy risk factors for non-consolidation [38,39]. In addition, diabetes can impair the healing process of the fracture. Patients receiving adequate insulin therapy have a lower risk of delayed and non-consolidation [40,41].

Several risk factors are closely linked to the type of fracture, such as the mechanism of injury as high or low energy trauma, vascular damage, and impairment of soft tissues. The number, the comminution of the fragments and the extent of the interfragmentary gap affect the healing process [42].

In light of such findings, all factors related to the patient and the fracture’s type need to be thoroughly accounted for during the forensic evaluation of unsuccessful femoral shaft fracture treatment. Such factors often play a decisive role in the development of pseudarthrosis and can determine the negative outcomes of orthopaedic surgical treatments. At the same time, it will also be necessary to verify whether adequate biological therapies have been administered to stimulate the reparative process. Therefore, attention in the evaluation of any profiles of medical professional responsibility must be focused on the patients themselves, without being limited to an exclusive judgment on mechanical stability and correctness of internal fixation. The latter is necessary but not enough for a positive outcome of the treatment. We must also always consider the biological environment of the fracture. From 2020, moreover, the SarsCoV-2 pandemic has been disrupting the regular provision of clinical-instrumental controls, creating management difficulties not attributable to the individual surgeons, limited in part by the introduction of the applications of telemedicine which made it possible to overcome some organisational difficulties [42].

## 5. Conclusions

The literature herein analysed and the results obtained from the analysis of our sample of patients underline the importance of the anatomo-functional damage caused by a femoral shaft pseudarthrosis, which can determine a 3 or 4 disability level, with severe or total functional impotence on the injured lower limb.

For the treatment of femoral pseudarthrosis, an accurate assessment of the patient’s general condition, age, type of fracture and bone quality is essential to choosing the most suitable treatment to create the best possible conditions conducive to bone healing.

From a medico-legal perspective, it is apparent that chronic consolidation outcomes after revision surgery of femoral shaft PSA cannot be compared with the results after a consolidated femoral fracture with mild functional repercussions. The PSA of the femur recovered after a second osteosynthesis with bone grafts is burdened by a major anatomical and functional impairment. Such impairment is caused by the double surgical access, the second one wider and more complex; by a morphologically and biomechanically less valid callus than the first intention consolidation one; by mild and unavoidable angular or rotational bone deformities; by greater residual hypotonia; by more significant repercussions in joint functions linked to the long therapeutic path and the myotendinous scars, especially of the knee.

The physical damage resulting from the outcomes of femoral shaft PSA consolidated after a revision osteosynthesis treatment and the bone grafts, will be more substantial than the permanent sequelae following a femoral diaphyseal fracture consolidated ab initio. The presence of any limitations at the hip and/or knee range of motion, deformity and/or dysmetria will require an adequate medico-legal evaluation.

## Figures and Tables

**Table 1:** Patient demographics and applied treatments.

Patients with Femoral PSA (*n* = 16)			
**Mean Age (years)**	50 (12; 82)	**Side**	
**Sex**		Left	9
		Right	7
Male	10	**Open fractures (*n*)**	1
Female	6	**Fracture localization (*n*)**	
**High-energy trauma (*n*)**	16	Proximal third	8
**First implant rupture (*n*)**	7	Middle third	4
**Initial treatment (*n*)**		Distal third	4
Intramedullary nail	8	**PSA treatment (*n*)**	
Plate and screw	8	Plate + homologous bone graft	7
**Smokers (*n*)**	3	Plate + cortico-cancellous bone graft	3
**Plate + screw**	8	Intramedullary nail	2
**Follow-up (months)**	15.4 (range 6–38)	Prosthetic implantation	4

## Data Availability

The data presented in this case report are available on request from the corresponding author.
